# Bidirectional Associations of Awareness of Age-Related Change and Attitudes Toward Own Aging With Social Media Use

**DOI:** 10.1093/geronb/gbad070

**Published:** 2023-05-13

**Authors:** Serena Sabatini, Bethany Wilton-Harding, Clive Ballard, Helen Brooker, Anne Corbett, Adam Hampshire, Tim D Windsor

**Affiliations:** School of Medicine, University of Nottingham, Nottingham, UK; College of Nursing and Health Sciences, Flinders University, Adelaide, Australia; University of Exeter Medical School, Exeter, UK; University of Exeter Medical School, Exeter, UK; Ecog Pro Ltd, Bristol, UK; University of Exeter Medical School, Exeter, UK; Social, Genetic, and Developmental Psychiatry Centre, King’s College London, London, UK; Social Sciences, Flinders University, Adelaide, South Australia, Australia

**Keywords:** Gains and losses, Middle and older age, Self-perceptions of aging, Technology use, Views on aging

## Abstract

**Objectives:**

We test whether higher awareness of age-related gains (AARC-gains), lower awareness of age-related losses (AARC-losses), and more positive attitudes toward own aging (ATOA) are cross-sectionally related to more frequent social media use. We also investigate the strength and direction of the associations of AARC-gains, AARC-losses, and ATOA with social media use over 1 year, from before to after the onset of the coronavirus 2019 pandemic.

**Methods:**

We used cross-sectional data from 8,320 individuals (mean age = 65.95 years; standard deviation = 7.01) and longitudinal data from a subsample of 4,454 individuals participating in the UK PROTECT study in 2019 and 2020. We used ordered regression models, linear regression models, and tests of interaction. Models were adjusted for age, sex, education, and employment.

**Results:**

Higher AARC-gains and more positive ATOA, but not AARC-losses, were cross-sectionally associated with more frequent social media use. Social media use became more frequent at follow-up. In the longitudinal models controlling for baseline levels of the outcome variable, more frequent baseline social media use predicted increases in AARC-gains, whereas baseline AARC-gains did not significantly predict the frequency of social media use at follow-up. Baseline frequency of social media use did not significantly predict AARC-losses, nor ATOA at follow-up, whereas lower levels of AARC-losses and more positive ATOA predicted more frequent social media use at follow-up.

**Discussion:**

Although effect sizes were small, decreasing negative views on aging may help increase the engagement of middle-aged and older people with social media. At the same time, fostering social media use could promote positive self-perceptions of aging.

Researchers are increasingly interested in the potential for social media use to play a role in supporting social engagement in later life. Although social media use is increasing, it remains less common among middle-aged and older adults, relative to younger adults (Cotten et al., 2021; Sala et al., 2022). According to a recent study from the Pew Research Center, in the United States over 80% of adults aged 18–49 years reported using social media, with this figure dropping to 73% among those aged 50–64 years, and to 45% among those aged 65+ (Auxier & Anderson, 2021). In the United Kingdom, it is estimated that 52% of individuals aged 45–54 years, 36% of individuals aged 55–64 years, and 16% of individuals aged 65+ use social media, respectively (Ofcom, 2022).

## Benefits of Social Media Use

Social media use can benefit multiple domains of middle-aged and older people’s lives. First, social media is seen as a tool that can strengthen social networks and intergenerational connectedness, and provide important information about social and community activity and events (Sala et al., 2022). Using social media to foster online (and offline) relationships could also serve as an important compensatory function for maintaining social connections in the context of less proximal networks and declining health in later life (Hülür & Macdonald, 2020). Second, social media use can be a means of acquiring and disseminating information across multiple societal domains including commerce, science, entertainment, politics, and crisis management (Hruska & Maresova, 2020). Third, the skills required to use social media and its evolving features, the information that can be acquired through social media, and the social interactions that media platforms make possible could each contribute to the maintenance and enhancement of cognitive functioning (Newman et al., 2021). Overall, research to date points to social media use being correlated with positive mental health and well-being outcomes among middle-aged and older adults (Chen et al., 2022; Cotten et al., 2021; Khoo & Yang, 2020).

The potential for social media use to foster and facilitate social connections and information acquisition and dissemination independently of time and geographical location has been brought into sharp focus by the coronavirus 2019 (COVID-19) pandemic. Both voluntary social distancing and enforced lockdowns have created challenges in maintaining social relationships that have persisted to varying degrees during the pandemic. Use of technology to maintain connections between and within generations had been both advocated by researchers (Moore & Hancock, 2020; Wu, 2020; Xie et al., 2020) and identified as a source of social exclusion among groups of adults where the use of technology and social media was/is low (Seifert et al., 2021).

Nonusers of social media cite concerns around privacy, a lack of interest, and/or the perception that online social connections are superficial and much of the content trivial as reasons for not engaging (Jung et al., 2017; Newman et al., 2021; Ofcom, 2022). Nonetheless, during the COVID-19 pandemic, social media use increased across all age groups (Aggarwal et al., 2022). It is therefore likely that, although many middle-aged and older people prefer in-person social interaction to online interaction when in-person interactions are not possible, a proportion of middle-aged and older people are willing and able to adapt to alternative ways of social interaction (i.e., virtual social interaction through the use of social media).

## Downsides of Social Media Use

Social media use, however, also has downsides including potential for harmful information to be shared, cyberbullying, and the disclosure and misuse of personal information (Leist, 2013). Moreover, problematic social media use (indicative of psychological dependence) has been associated with social isolation in older adults (Meshi et al., 2020). In addition, social media can also provide a platform for overt ageism and intergenerational conflict; this was particularly the case during early stages of the COVID-19 pandemic (Jimenez-Sotomayor et al., 2020; Meisner, 2021; Soto-Perez-de-Celis, 2020; Xiang et al., 2021) when, for example, the hashtag “*#BoomerRemover*” gained traction on Twitter. Qualitative studies examining the content of English-language tweets about COVID-19 and older adults found that between 12% and 14% of Tweets posted at the beginning of the COVID-19 pandemic contained offensive content or jokes against older people (Jimenez-Sotomayor et al., 2020; Xiang et al., 2021). It is therefore plausible that social media use during the COVID-19 pandemic has not only led to benefits but also to potential harm to the psychological well-being of older people. For example, research studies showed that the way in which older people perceived themselves became more negative since the start of the COVID-19 pandemic (Ayalon, 2020; Fernandes Silva et al., 2021; Kornadt et al., 2021; Previtali et al., 2020; Swift & Chasteen, 2021).

## Factors Related to Social Media Use in Middle and Older Age

Overall, it seems likely that for many middle-aged and older adults—particularly those who may be socially isolated or have restricted social networks—social media use has the potential to facilitate meaningful social engagement and to be a useful source of information. It is therefore important to consider the personal and contextual factors that could predict social media use. One recent population-based study of older adults in Finland found that women and those with more education and more children were more likely to use social media (Tammisalo et al., 2022). In addition to sociodemographic factors, researchers have also focused on middle-aged and older adults’ attitudes toward social media and other personal attributes as predictors of social media and technology use. For example, a study of 124 internet-using adults aged 60–90 years found that perceived usefulness, trust in social networking sites, and frequency of internet use predicted social media use (Braun, 2013). Another factor that may be related to social media use is people’s views of their own aging.

Views on aging (VoA) refer to people’s beliefs about age, aging, and older people in relation to themselves and others (Kornadt et al., 2019). VoA encompasses various constructs such as subjective age (Barrett, 2003), attitudes toward own aging (ATOA; Lawton, 1975), and awareness of age-related change (AARC; Diehl & Wahl, 2010; Diehl et al., 2014). VoA has been consistently associated with a range of consequential outcomes for development including social engagement (Schwartz et al., 2021), mental health (Sabatini et al., 2020), cognitive functioning (Sabatini et al., 2021; Stephan et al., 2014), and physical health and mortality (Westerhof et al., 2014).

Emerging research also points to links between VoA and technology use. A younger subjective age was found to be associated with greater internet use (Seifert et al., 2021) and a greater ability to use technologies (Sghaier et al., 2022). Nonetheless, Wan et al. (2022) found no association between changes in subjective age and changes in internet use over 8 years. Finally, Schlomann et al. (2022) showed that greater awareness of age-related gains (AARC-gains) was cross-sectionally associated with more positive attitudes toward technology and greater technology skills, whereas greater awareness of age-related losses (AARC-losses) was associated with more negative attitudes, poorer skills, and less general technology use in individuals aged 65–93 years.

## Theory and Evidence in Support of Bidirectionality Between Social Media Use and VoA

Bidirectional processes involving social media use and VoA are plausible. Conceptual perspectives on VoA offer several reasons why people’s VoA could affect social media use. First, stereotype embodiment theory (Levy, 2009) identifies processes through which individuals begin to see culturally accepted negative stereotypes about aging as self-relevant as they themselves grow older. Thus, older individuals who hold negative VoA may see themselves as less capable of successfully surmounting challenges around learning to use social media (Jung et al., 2017).

Second, the experience and consequent realization of age-related gains could facilitate adaptation to social media use. Indeed, according to Diehl and Wahl (2010), AARC may contribute to effective self-regulatory behaviors, and the flexible management of goals around engaging with new technologies is likely to facilitate adaptation. Third, consistent with Baltes’ Selection, Optimization, and Compensation model (Baltes & Baltes, 1990) in the context of physically impairing health conditions that restrict mobility and make in-person social interactions more difficult and less frequent (thereby highlighting AARC-losses), individuals may opt for social media use as a compensatory means to maintain social connections.

Similarly, in the context of the COVID-19 pandemic, and the consequent impossibility to have in-person social interactions, some of those middle-aged and older people who were not already using social media, may have decided to start using them to maintain social interactions, and may have directed significant time and energy resources (Huxhold et al., 2022) toward learning how to use social media. Those individuals who had more positive VoA, were more aware of positive age-related changes (e.g., accumulated knowledge), and/or perceived less age-related losses (e.g., perceived decreased cognitive capacity) may have been particularly better placed to select and successfully pursue goals related to adopting and learning social media use.

Social media use may also influence VoA. For example, older adults who successfully use social media may see themselves as remaining “in-touch” with the rapidly changing information technology landscape contributing to positive views of one’s own aging. Although there is no specific research on social media use, studies focusing more broadly on technology use and VoA suggest that social media use could enhance positive VoA. For example, Köttl et al. (2021) found that technology use predicted subsequent perceptions of personal competence. Literature linking positively valued behaviors (e.g., physical activity) with VoA also suggests that people’s behaviors can increase their positive VoA (Brothers & Diehl, 2017; Klusmann et al., 2012). Moreover, the wide amount of evidence highlighting the benefits of social media on people’s health and well-being (Chen et al., 2022; Cotten et al., 2021; Khoo & Yang, 2020) suggests it is plausible that these benefits may be extended to the way in which middle-aged and older people view themselves. In contrast, negative experiences with social media due to a lack of skills undermining self-efficacy, or arising from exposure to ageist content, may subsequently make middle-aged and older people feel more negatively about themselves and their aging (Köttl et al., 2021). For example, one recent study found that older adults felt older after engaging with applications on a tablet, particularly when those applications were unfamiliar (Caspi et al., 2019).

## The Current Study

Overall, key developmental theories and available evidence on technology use in relation to VoA, suggest that more positive and less negative VoA may facilitate social media use (Schlomann et al., 2022; Seifert et al., 2021). At the same time, depending on the individual experience of social media, these could either highlight age-related limitations (i.e., AARC-losses; Caspi et al., 2019; Sghaier et al., 2022) or lead to positive feelings and positive self-perceptions (i.e., AARC-gains and more positive ATOA). The present study examines associations of AARC-gains and AARC-losses and ATOA with social media use, using two waves of data collected in 2019 and 2020 as part of the UK PROTECT cohort study. We focus on the relationship between social media use (as opposed to technology use more generally) and VoA, due to the lack of previous research in the area, the proposed utility of social media use as a compensatory tool for maintaining social networks (Hülür & Macdonald, 2020), and the fact that the UK PROTECT study was conducted online, which suggests that all participants would have had at least basic familiarity with technology in order to participate in the study.

Our research builds on the existing literature in several important ways. In addition to replicating the examination of cross-sectional associations of AARC with an aspect of technology use (i.e., social media; e.g., Schlomann et al., 2022) in a large sample, we examine possible bidirectional associations of VoA with subsequent social media use and vice versa. Here we extend recent longitudinal work by Köttl et al. (2021) by examining separate domains of VoA (i.e., AARC-gains and AARC-losses) and by focusing on social media use as a separate aspect from everyday technologies used. Second, the timing of data collection meant that a subsample of participants provided data both before (baseline in 2019) and after (follow-up in 2020) the onset of the COVID-19 pandemic. This provides a unique opportunity to examine for the first time the impact of this key history-graded event on middle-aged and older adults’ degree of social media use, and how this relates to VoA.

In the current study, first, we aim to explore whether higher AARC-gains, lower AARC-losses, and more positive ATOA are related to more frequent social media use in our larger cross-sectional sample in 2019 (prior to the COVID-19 pandemic). Based on previous research on technology use (Schlomann et al., 2022; Seifert et al., 2021) we expect that more positive VoA (i.e., higher AARC-gains, lower AARC-losses, more positive ATOA) are associated with more frequent social media use. Second, we aim to investigate the strength and direction of the associations of AARC-gains, AARC-losses, and ATOA with changes in frequency of social media use over 1 year. Given the plausibility of reciprocal associations (Köttl et al., 2021) we predict that relatively more negative VoA (i.e., lower AARC-gains, higher AARC-losses, and more negative ATOA) is associated with less increase in social media use from pre- to postonset of the pandemic. At the same time, we expect that lower social media use pre-pandemic is associated with less positive (or more negative) changes in VoA over the study interval.

## Method

### Study Design

This study uses data collected online through the UK PROTECT (Platform for Research Online to investigate Genetics and Cognition in Ageing) cohort study (https://www.protectstudy.org.uk) in 2019 and 2020. Individuals can enroll in the UK PROTECT study if they are UK resident, English speaker, aged 50+, have access to the internet, and lack of clinical diagnosis of dementia at baseline. During recruitment, which started in 2015, the study was publicized nationwide and among existing research cohorts of middle-aged and older adults including *Exeter 10,000*, *Join Dementia Research*, and *Brains for Dementia Research*. At baseline (2015), participants provided informed consent online. UK PROTECT obtained ethical approval from the London Bridge NHS Research Ethics Committee and Health Research Authority (Ref:13/LO/1578).

UK PROTECT participants are invited to take part in a follow-up assessment each year. As part of their annual assessments in 2019 and 2020, participants were asked to complete additional questions assessing AARC-gains, AARC-losses, and ATOA. A total of 8,320 participants completed questions of AARC, ATOA, and social media use in 2019 and comprised the cross-sectional analytical sample. Of these, 4,454 competed the same measures again in 2020 and comprised the longitudinal analytical sample for this study.

### Measures

#### Sociodemographic variables

Sociodemographic variables comprised age, sex, education (*secondary education; postsecondary education; vocational qualifications; undergraduate degrees; postgraduate degrees; doctorates*); and employment status (*employed; not employed*).

#### Awareness of age-related change

AARC was measured using the 10-item version of the questionnaire (AARC-10 SF; Kaspar et al., 2019) comprising five items assessing AARC-gains and five items assessing AARC-losses. An item in each of the AARC-gains and AARC-losses subscales represents one of the five AARC life and behavioral domains. Each item starts with the stem: “With my increasing age, I realize that….” Respondents rate how much items apply to them (1 = not at all; 2 = a little bit; 3 = moderately; 4 = quite a bit; 5 = very much). Scores are obtained for the AARC-gains and AARC-losses subscales by summing the five items within the respective subscales. Higher scores indicate higher AARC-gains/losses (range: 5–25). In this sample, Cronbach’s alpha for internal consistency for the AARC-gains subscale was 0.76 and for the AARC-losses subscale was 0.79.

#### Attitudes toward own aging

ATOA were measured using the five-item subscale from the Philadelphia Geriatric Center Morale Scale (Lawton, 1975). For each statement, respondents are asked to make temporal comparisons about changes in energy level, perceived usefulness, happiness, and quality of life and to respond on a binary response set (*better* vs *worse*, *yes* vs *no*). An example item is “things keep getting worse as I get older.” We used a proportion-based score obtained by summing participant’s item scores and dividing it by the number of responses, producing scores ranging from 0 to 1. In this sample, Cronbach’s alpha for internal consistency for the ATOA scale was 0.85.

### Social Media Use

Social media use was assessed with the question “How often do you use…? Social media (Facebook, Twitter, Instagram, Whatsapp, etc.).” Participants indicated how frequently they engage with social media (0 = *never*; 1 = *occasionally*; 2 = *once a month*; 3 = *once a week,* 4 = *once a day*; 5 = *more than once a day*).

### Analyses

We reported summary statistics for study variables in 2019 (baseline for this study) for the overall cross-sectional analytical sample (Table 1). We also reported summary statistics at baseline and 1-year follow-up for the longitudinal analytical sample. Moreover, we reported summary statistics and statistical differences for study variables at baseline for the longitudinal analytical sample and for those who did not provide longitudinal data (Supplementary Table 1).

**Table 1.** Descriptive Statistics for Study Participants

	Cross-sectional study sample, *N* = 8,320	Longitudinal study sample, *N* = 4,454
Age; *M* (*SD*; range)	65.95 (7.01; 52–95)	65.96 (6.85; 51–95)
Women, *n* (%)	6,470 (77.8)	3,475 (78.0)
Education, *n* (%)		
Secondary education	1,130 (13.58)	592 (13.3)
Postsecondary education	961 (11.6)	514 (11.4)
Vocational qualification	1,644 (19.8)	899 (20.2)
Undergraduate degree	2,791 (33.6)	1,516 (34.0)
Postgraduate degree	1,479 (17.8)	765 (17.2)
Doctorate	315 (3.8)	168 (3.8)
Employed, *n* (%)	3,393 (41.8)	1,737 (40.0)
Baseline awareness of age-related gains, *M* (*SD*)	17.93 (3.91)	18.00 (3.89)
Follow-up awareness of age-related gains, *M* (*SD*)		18.16 (8.81)
Baseline awareness of age-related losses, *M* (*SD*)	9.88 (3.25)	9.76 (3.20)
Follow-up awareness of age-related losses, *M* (*SD*)		10.16 (3.40)
Baseline attitudes toward own aging, *M* (*SD*)	0.68 (0.19)	0.68 (0.19)
Follow-up attitudes toward own aging, *M* (*SD*)		0.67 (0.20)
Baseline frequency of social media use, *n* (%)		
Never	1,269 (15.3)	707 (15.9)
Occasionally	997 (12.0)	535 (12.09)
Once a month	54 (0.7)	31 (0.7)
Once a week	517 (6.2)	308 (6.9)
Once a day	1,763 (21.2)	960 (21.6)
More than once a day	3,720 (44.7)	1,913 (43.0)
Follow-up frequency of social media use, *n* (%)		
Never		499 (11.2)
Occasionally		423 (9.5)
Once a month		18 (0.4)
Once a week		218 (4.9)
Once a day		1,100 (24.7)
More than once a day		2,196 (49.3)

*Notes:* The cross-sectional analytical sample comprises those participants who reported data on awareness of age-related changes and use of technologies in 2019 (baseline). The longitudinal analytical sample comprises a subsample of participants who reported data on awareness of age-related changes and use of technologies both in 2019 and 2020 (follow-up). *SD* = standard deviation.

To investigate the cross-sectional associations of AARC-gains, AARC-losses, and ATOA (predictors) with frequency of social media use (outcome) we fitted ordinal regression models using the *Oprobit* function in STATA. As the sample comprised middle-aged, early old, and advanced old individuals (age range 50–95 years), we used tests of interaction to examine whether the interaction between each of AARC-gains, AARC-losses, and ATOA with age as a categorical variable (midlife = 50–64 years, early old age = 65–74 years, and advanced old age = 75+ years) explained variance in frequency social media use at cross-sectional level. Significance for the test of interaction was set at the 5% level.

To examine longitudinal associations, we fit ordered lagged regression models to explore the influences of AARC-gains, AARC-losses, and ATOA on frequency of social media use over 1 year (using two timepoints) via the STATA *Oprobit* function (Newsom, 2013). Each model comprised baseline scores on AARC-gains, AARC-losses, or ATOA and on social media use as predictors of social media use at follow-up. We fit linear regression models to explore the influence of baseline frequency of social media use and baseline VoA (either baseline AARC-gains, baseline AARC-losses, or baseline ATOA) on follow-up VoA (either AARC-gains, AARC-losses, or ATOA).

For all regression models we fitted both an unadjusted and an adjusted (for age, sex, education, and employment status) model. Complete case analyses were conducted. Analyses were conducted in STATA version 17.

## Results

### Study Sample

The cross-sectional analytical sample comprised 8,320 individuals aged on average 65.95 years. At baseline, the majority of participants were women, and the majority had obtained a university degree. Participants reported moderate levels of awareness of age-related gains, low levels of awareness of age-related losses, and mostly positive attitudes toward own aging. Although about 85% reported using social media, the frequency of social media use varied greatly among participants. The longitudinal analytical sample comprised 4,454 participants. Their mean scores on study variables at baseline were similar to those of the cross-sectional analytical sample. In this longitudinal subsample, mean scores for both AARC-gains and AARC-losses slightly increased from baseline to follow-up assessments whereas mean scores on ATOA minimally decreased (i.e., ATOA became more negative). The average frequency of social media use increased between 2019 and 2020.

Descriptive statistics for the cross-sectional and longitudinal analytical samples are reported in Table 1. Supplementary Table 1 reports descriptive statistics at baseline for the longitudinal analytical sample and for those who did not provide longitudinal data. Compared to the longitudinal analytical sample, participants who did not provide longitudinal data were marginally more likely to be employed and to use social media. They also reported marginally higher AARC-losses.

### Cross-Sectional Associations of VoA With Social Media Use

Table 2 reports the results of ordinal regression models with AARC-gains, AARC-losses, and ATOA as cross-sectional predictors of frequency of social media use, as well as tests of interactions examining whether the interaction between each of AARC-gains, AARC-losses, and ATOA and age groups explains variance in social media use. In the regression models adjusted for age, sex, education, and employment status, higher AARC-gains were cross-sectionally associated with greater social media use. AARC-losses and ATOA were instead not significantly associated with social media use at cross-sectional level.

**Table 2.** AARC-Gains, AARC-Losses, and ATOA as Cross-Sectional Predictors of Frequency of Social Media Use

	Social media use					
	Unadjusted model			Adjusted model		
	Beta (95% CI)	Odd ratios (95% CI)	Pseudo *R*^2^	Beta (95% CI)	Odd ratios (95% CI)	Pseudo *R*^2^
AARC-gains	0.04 (0.03; 0.04)***	1.04 (1.03; 1.04)***	1%	0.03 (0.02; 0.04)***	1.03 (1.02; 1.04)***	1%
Age groups						
Midlife	(Reference)	(Reference)		(Reference)	(Reference)	
Early old age	−0.45 (−0.68; −0.21)***	0.64 (0.51; 0.82)***		−0.32 (−0.56; −0.07)*	0.73 (0.57; 0.93)*	
Advanced old age	−1.04 (−1.43; −0.65)***	0.35 (0.24; 0.52)***		−0.87 (−1.26; −0.47)***	0.42 (0.28; 0.63)***	
AARC-gains × age groups						
Midlife	(Reference)	(Reference)		(Reference)	(Reference)	
Early old age	0.002 (−0.01;.01)	1.002 (0.99; 1.01)		0.0002 (−0.01; 0.01)	1.002 (0.99; 1.01)	
Advanced old age	−0.0004 (−0.02;.02)	1.00 (0.98; 1.02)		−0.002 (−0.02; 0.02)	1.00 (0.98; 1.02)	
AARC-losses	−0.03 (−0.03; −0.02)***	0.97 (0.97; 0.98)***	0.2%	0.002 (−0.01; 0.01)	1.002 (0.99; 1.01)	0
Age groups						
Midlife	(Reference)	(Reference)		(Reference)	(Reference)	
Early old age	−0.30 (−0.47; −0.14)***	0.74 (0.63; 0.87)***		−0.23 (−0.40; −0.06)**	0.79 (0.67; 0.94)**	
Advanced old age	−0.64 (−0.92; −0.37)***	0.53 (0.40; 0.69)***		−0.54 (−0.83; −0.26)***	0.58 (0.44; 0.77)***	
AARC-losses × age groups						
Midlife	(Reference)	(Reference)		(Reference)	(Reference)	
Early old age	−0.01 (−0.03; 0.004)	0.99 (0.97; 1.004)		−0.01 (−0.03; 0.01)	0.99 (0.97; 1.01)	
Advanced old age	−0.04 (−0.06; −0.01)**	0.096 (0.94; 0.99)**		−0.03 (−0.06; −0.01)**	0.97 (0.94; 0.99)**	
ATOA	0.45 (0.33; 0.58)***	1.57 (1.39; 1.79)	0.2%	0.11 (−0.02; 0.24)	1.11 (0.98; 1.27)	0
Age groups						
Midlife	(Reference)	(Reference)		(Reference)	(Reference)	
Early old age	−0.55 (−0.75; −0.35)***	0.58 (0.47; 0.70)***		−0.43 (−0.64; −0.22)***	0.65 (0.53; 0.80)***	
Advanced old age	−1.32 (−1.58; −1.07)***	0.27 (0.21; 0.14)***		−1.16 (−1.42; −0.89)***	.31 (0.24; 2.44)***	
ATOA × age groups						
Midlife	(Reference)	(Reference)		(Reference)	(Reference)	
Early old age	0.19 (−0.09; 0.47)	1.21 (0.91; 1.60)		0.15 (−0.13; 0.44)	1.16 (0.88; 1.55)	
Advanced old age	0.45 (0.07; 0.84)*	1.57 (1.07; 2.32)*		.41 (0.02; 0.80)*	1.51 (1.02; 2.23)*	

*Notes: N* = 8,320. Adjusted for age, sex, education, and employment status. AARC = awareness of age-related change; ATOA = attitudes toward own aging; CI = confidence interval.

***p < .001. **p < .01. *p < .05.

The interaction between AARC-gains and age group was not a statistically significant predictor of social media use. The interactions of AARC-losses and ATOA with age group as predictors of social media use showed that, compared to those in midlife, the association between more negative VoA and less social media use was significantly stronger in advanced old age, but not in early old age.

### Bidirectional Associations of VoA With Social Media Use

Tables 3–5 report the results of ordinal regression models exploring the influence of baseline AARC-gains, baseline AARC-losses, and baseline ATOA on follow-up frequency of social media use while controlling for baseline levels of the outcome and demographic variables. Tables 3–5 also report results of linear regression models exploring the influence of baseline social media use on follow-up AARC-gains, AARC-losses, and ATOA while controlling for baseline levels of the outcome and demographic variables. We found that more frequent social media use at baseline predicted increases in AARC-gains but AARC-gains at baseline did not predict change in social media use. Frequency of social media use did not significantly predict changes in AARC-losses, nor ATOA, whereas lower AARC-losses and more positive ATOA predicted increases in social media use from baseline to follow-up.

**Table 3.** Bidirectional Associations of AARC-Gains With Frequency of Social Media Use

Outcomes	Predictors	Unadjusted model		Adjusted model	
		Beta (95% CI)	Odd ratios (95% CI)	Beta (95% CI)	Odd ratios (95% CI)
Follow-up AARC-gains					
	Baseline AARC-gains	0.59 (0.57; 0.61)***	1.80 (1.77; 1.84)***	0.58 (0.56; 0.60)***	1.79 (1.75; 1.82)***
	Baseline frequency of social media use (reference: never)				
	Occasionally	0.23 (−0.06; 0.52)	1.26 (0.94; 1.68)	0.27 (−0.02; 0.57)	1.31 (0.98; 1.77)
	Once a month	0.06 (−0.88; 1.00)	1.06 (0.41; 2.72)	0.06 (−0.88; 1.00)	1.06 (0.41; 2.72)
	Once a week	0.16 (−0.19; 0.51)	1.17 (0.83; 1.66)	0.23 (−0.13; 0.58)	1.26 (0.88; 1.79)
	Once a day	0.38 (0.13; 0.63)**	1.46 (1.14; 1.88)**	0.43 (0.17; 0.69)**	1.54 (1.19; 1.99)**
	More than once a day	0.28 (0.05; 0.50)*	1.32 (1.05; 1.65)*	0.35 (0.11; 0.58)**	1.42 (1.12; 1.79)**
Follow-up frequency of social media use					
	Baseline AARC-gains	0.01 (0.001; 0.02)*	1.01 (1.001; 1.01)*	0.01 (−0.002; 0.02)	1.01 (1.00; 1.02)
	Baseline frequency of social media use (reference: never)				
	Occasionally	1.03 (0.91; 1.16)***	2.80 (2.48; 3.19)***	1.02 (0.90; 1.15)***	2.88 (2.46; 3.16)***
	Once a month	1.12 (0.75; 1.49)***	3.06 (2.12; 4.44)***	1.09 (0.72; 1.47)***	2.97 (2.05; 4.35)***
	Once a week	1.45 (1.30; 1.59)***	2.26 (3.67; 4.90)***	1.40 (1.25; 1.55)***	4.06 (3.49; 4.71)***
	Once a day	1.89 (1.77; 2.00)***	6.62 (5.87; 7.39)***	1.82 (1.70; 1.94)***	6.17 (5.47; 6.96)***
	More than once a day	2.86 (2.74; 2.97)***	17.46 (15.49; 19.49)***	2.78 (2.66; 2.90)***	16.12 (14.30; 18.17)***

*Note:* Models are adjusted for age, sex, education, and employment status. AARC = awareness of age-related change; CI = confidence interval.

***p < .001. **p < .01. *p < .05.

**Table 4.** Bidirectional Association of AARC-Losses With Frequency of Social Media Use.

Outcomes	Predictors	Unadjusted model		Adjusted model	
		β (95% CI)	Odd ratios (95% CI)	β (95% CI)	Odd ratios (95% CI)
Follow-up AARC-losses					
	Baseline AARC-losses	0.78 (.76; 0.80)***	2.18 (2.14; 2.23)***	0.75 (.73; 0.77)***	2.12 (2.08; 2.16)***
	Baseline frequency of social media use (reference: never)				
	Occasionally	−0.26 (−0.47; −0.04)*	0.77 (0.63; 0.96)*	−0.18 (−0.40; 0.04)	0.84 (0.67; 1.04)
	Once a month	−0.03 (−0.74; 0.69)	0.97 (0.48; 1.99)	−0.09 (−0.62; 0.81)	0.91 (0.54; 2.25)
	Once a week	−0.42 (−0.69; −0.15)**	0.66 (0.50; 0.86)**	−0.24 (−0.51; 0.03)	0.79 (0.60; 1.03)
	Once a day	−0.33 (−0.52; −0.14)**	0.72 (0.59; 0.87)**	−0.17 (−0.37; 0.02)	0.84 (0.69; 1.02)
	More than once a day	−0.40 (−0.57; −0.23)***	0.67 (0.57; 0.79)***	−0.14 (−0.32; 0.04)	0.87 (0.73; 1.04)
Follow-up frequency of social media use					
	Baseline AARC-losses	−0.03 (−0.04; −0.02)***	0.97 (0.96; 0.98)***	−0.02 (−0.03; −0.01)***	0.98 (0.97; 0.99)***
	Baseline frequency of social media use (reference: never)				
	Occasionally	1.04 (.92; 1.16)***	2.83 (2.52; 3.19)***	1.03 (0.91; 1.16)***	2.80 (2.48; 3.19)***
	Once a month	1.12 (.74; 1.49)***	3.06 (2.10; 4.44)***	1.10 (0.72; 1.47)***	3.00 (2.05; 4.35)***
	Once a week	1.45 (1.30; 1.59)***	4.26 (3.67; 4.90)***	1.41 (1.25; 1.56)***	4.10 (3.49; 4.76)***
	Once a day	1.89 (1.77; 2.00)***	6.62 (5.87; 7.39)***	1.83 (1.71; 1.95)***	6.23 (5.53; 7.03)***
	More than once a day	2.87 (2.75; 2.98)***	17.64 (15.64; 19.69)***	2.79 (2.67; 2.91)***	16.28 (14.44; 18.36)***

*Notes:* Models are adjusted for age, sex, education, and employment status. AARC = awareness of age-related change; CI = confidence interval.

***p < .001. **p< .01. *p < .05.

**Table 5.** Bidirectional Association of ATOA With Frequency of Social Media Use.

Outcomes	Predictors	Unadjusted model		Adjusted model	
		β (95% CI)	Odd ratios (95% CI)	β (95% CI)	Odd ratios (95% CI)
Follow-up ATOA					
	Baseline ATOA	0.56 (0.53; 0.58)***	1.75 (1.70; 1.79)***	0.53 (0.51; 0.56)***	1.70 (1.67; 1.75)
	Baseline frequency of social media use (reference: never)				
	Occasionally	0.01 (−0.01; 0.02)	1.01 (0.99; 1.02)	−0.001 (−0.02; 0.02)	1.00 (0.98; 1.02)
	Once a month	0.05 (−0.004; 0.11)	1.05 (1.00; 1.12)	0.04 (−0.02; 0.09)	1.04 (0.98; 1.09)
	Once a week	0.02 (−0.004; 0.04)	1.02 (1.00; 1.04)	0.003 (−0.02; 0.02)	1.00 (0.98; 1.02)
	Once a day	0.02 (0.01; 0.03)**	1.02 (1.01; 1.03)**	0.01 (−0.01; 0.02)	1.01 (0.99; 1.02)
	More than once a day	0.02 (0.01; 0.04)***	1.02 (1.01; 1.04)***	0.01 (−0.01; 0.02)	1.01 (0.99; 1.02)
Follow-up frequency of social media use					
	Baseline ATOA	0.45 (0.26; 0.63)***	1.57 (1.30; 1.88)***	0.35 (0.15; 0.54)***	1.42 (1.16; 1.72)***
	Baseline frequency of social media use (reference: never)				
	Occasionally	1.03 (0.91; 1.15)***	2.80 (2.48; 3.16)***	1.02 (0.90; 1.15)***	2.77 (2.46; 3.16)***
	Once a month	1.13 (0.76; 1.51)***	3.10 (2.14; 4.53)***	1.11 (0.73; 1.48)***	3.03 (2.08; 4.39)***
	Once a week	1.43 (1.28; 1.58)***	4.18 (3.60; 4.85)***	1.39 (1.24; 1.54)***	4.01 (3.46; 4.66)***
	Once a day	1.88 (1.77; 2.00)***	6.55 (5.87; 7.39)***	1.82 (1.70; 1.94)***	6.17 (5.47; 6.96)***
	More than once a day	2.86 (2.74; 2.97)***	17.46 (15.49; 19.49)***	2.79 (2.67; 2.91)***	16.28 (14.44; 18.36)***

*Note:* Models are adjusted for age, sex, education, and employment status. AARC = awareness of age-related change; ATOA = attitudes toward own aging; CI = confidence interval.

***p < .001. **p < .01.

## Discussion

This study examined cross-sectional and bidirectional longitudinal associations between indicators of VoA and social media use. Cross-sectionally, individuals with higher AARC-gains reported more frequent social media use whereas AARC-losses and ATOA were not significantly associated with the frequency of social media use. Longitudinal analyses showed that lower AARC-losses and more positive ATOA at baseline predicted increases in social media use over 1 year. In addition, more frequent social media use at baseline predicted increases in AARC-gains over 1 year. However, associations were small. Nonetheless, the different pattern of results we observed across indicators of VoA suggests that while decreasing negative self-perceptions of aging may be most important to promote social media use, fostering social media use could potentially lead to increased AARC-gains.

Our cross-sectional finding that individuals with greater AARC-gains used social media more frequently was consistent with both our predictions and previous studies showing that more positive VoA is associated with more positive attitudes toward technology (Schlomann et al., 2022), greater technology skills (Schlomann et al., 2022), and social connectedness (Menkin et al., 2017). Our prediction that lower social media use would correlate with higher AARC-losses and more negative ATOA was not supported. However, considering age as a moderator showed that these associations were statistically reliable and in the expected direction among the subgroup of participants in advanced old age. This may be due to negative VoA being more salient in older age thereby increasing variability and the ability to detect significant associations. These findings raise the possibility that fostering social media use could promote more positive VoA in advanced old age.

Frequency of social media use increased over the 1-year study period. This is likely a compensatory response to lockdowns and social distancing from pre- to postonset of the pandemic. Our results, suggesting that social media use becomes more frequent when middle-aged and older people cannot leave their house, could be extended to other situations when older people cannot go outside and meet people as frequently as before (e.g., due to mobility problems). In these cases, social media use may help to maintain social connection, which is fundamental for maintenance of well-being and good quality of life throughout all phases of adulthood (Carstensen, 1993; Lindsay-Smith et al., 2018).

Our results suggest that older people who reported greater engagement with social media at baseline consequently reported more positive changes in AARC-gains. According to the Selection, Optimization, and Compensation theory (Baltes, 1997) social media use during the pandemic may represent an adaptive strategy that may enable middle-aged and older people to maintain social interactions in a period where previous resources are no longer available. Participants who were already engaged with social media prior to the onset of the pandemic may have been well placed to maintain and enhance positive VoA as they negotiated pandemic-related challenges. Our findings suggesting that older people can experience gains (i.e., learn a new strategy that in this case is social media use) when facing challenges are in line with previous evidence documenting how in middle and older age the experience of many gains can coexist with the experience of many losses (Sabatini et al., 2022). In addition, previous studies have shown that AARC is related to how people respond to change or challenges. For example, Dutt et al. (2016) found that developmental regulation was associated with higher AARC-gains and lower AARC-losses. Moreover, more positive VoA was found related to lower feelings of loneliness and greater health and well-being in older people during the pandemic (Avidor et al., 2021; Kornadt et al., 2021; Shrira et al., 2020).

It is also possible that those who already engaged with social media to a greater extent at baseline may have remained more connected to others (e.g., friends, children, grandchildren) and, thanks to positive social interactions, they may have experienced higher AARC-gains (O’Brien & Sharifian, 2019). These individuals may have also experienced increased feelings of competence (Deci & Ryan, 2012), self-efficacy (Bandura, 1977), and personal control over their own aging process, therefore fostering more positive perceptions of their own aging (Diehl & Wahl, 2010).

Some middle-aged and older people may be more predisposed to social media use than others due to individual differences (e.g., social media use is greater among women, people with better education, and people with more children), as previous evidence has shown (Tammisalo et al., 2022). Our results suggest that how older people view their own aging is an additional variable that influences whether and how often middle-aged and older people use social media. Indeed, our finding that higher AARC-losses at baseline predicted less uptake of social media use during the pandemic suggests that older people with more negative VoA may view their age as a barrier to learning a new skill, even when learning this skill could have led to better opportunities for remaining informed and socially connected during the COVID-19 pandemic.

This finding may be partially explained by stereotype embodiment theory, where individuals increasingly identify with negative societal aging stereotypes as they grow older. Older adults are often portrayed and perceived as being underconfident and underskilled in their use of technology (Kania-Lundholm & Torres, 2015). Hence, those with more negative ATOA and greater AARC-losses may be more easily discouraged in their efforts to engage with social media. Conversely, those who reported relatively lower AARC-losses and had more positive ATOA may be less likely to view their age as a barrier to gaining new skills (Schlomann et al., 2022).

Overall, our results suggest that future research focused on encouraging and facilitating engagement with social media may be worthwhile. Negative age-related stereotypes depicting older people as not being able to learn how to use technologies and social media are likely internalized by older people and, as our results suggest, this negative internalization could undermine efforts to engage with social media. There is the potential that decreasing negative age-related stereotypes and self-perceptions of aging may encourage engagement in social media and that targeting social media self-efficacy may improve positive self-perceptions of aging. Although this is a relatively new area, researchers have suggested that interventions should combat ageist stereotypes surrounding technology and aging (Köttl et al., 2021; Pruchno, 2019), foster lifelong learning (Köttl et al., 2021), and focus on the person as an individual, their social network, and their environment (König et al., 2018). As our results suggest that those with higher AARC-losses may be less prone to increase their engagement with social media, individuals with high AARC-losses may be the group who would benefit the most from interventions that promote the use of technologies and social media.

### Strengths and Limitations

This study had several strengths including the large sample representing both middle-aged and older individuals. Moreover, it was the first study exploring the bidirectional associations of AARC-gains and AARC-losses with social media use. In addition, although the study follow-up lasted only 1 year, it captured changes in frequency of social media use pre and during the COVID-19 pandemic where social media use became especially relevant as a tool for maintaining social connections.

Nonetheless, this study had important limitations. The first is selection bias. Due to the UK PROTECT study inclusion criterion, the UK PROTECT study sample is a cohort of participants already using technological devices provided with the internet. Hence, analyses are based on a selective sample of participants with a greater propensity toward the use of technologies. Results should, therefore, be generalized to the broader population with caution. Second, a significant proportion of participants was lost to follow-up; however, those who provided data at both timepoints did not meaningfully differ in study variables at baseline from those who provided data at baseline only. Moreover, in the UK PROTECT study, it is not mandatory for participants to complete all study measures at each timepoint.

## Conclusion

This paper demonstrated that indicators of VoA are related to how often individuals engage in social media. Social media use was more frequent among people who reported higher AARC-gains and more positive ATOA. Those who had more negative attitudes and perceptions about their aging at baseline reported less frequent social media use at follow-up. Finally, those who engaged with social media more frequently at baseline reported greater AARC-gains at follow-up. Although these findings are among the first to suggest potential links between VoA and social media use, associations over 1 year were of negligible-to-small size. Nonetheless, future research may benefit from evaluating personalized approaches to facilitate social media engagement among both middle-aged and older adults.

## Supplementary Material

gbad070_suppl_Supplementary_Tables

## Data Availability

UK PROTECT data are available upon request and approval from the UK PROTECT steering committee.

## Preregistration

Request to explore the associations of AARC-gains, AARC-losses, and ATOA with the use of technologies, the internet, and social media was presented to and approved by the PROTECT steering committee as part of a data request form.
