# Supplementary material for: Bidirectional Associations of Awareness of Age-Related Change and Attitudes Toward Own Aging With Social Media Use
Source: J Gerontol B Psychol Sci Soc Sci. 2023 May 13;78(8):1349–59. doi: 10.1093/geronb/gbad070 (PMC10394997; doi:10.1093/geronb/gbad070)
Supplement: gbad070_suppl_Supplementary_Tables [file gbad070_suppl_supplementary_tables.docx]

| **Supplementary Table 1.**  Descriptive Statistics for the Longitudinal Study Sample and Those Excluded From the Longitudinal Study Sample | | | | |
| --- | --- | --- | --- | --- |
|  | Longitudinal Study Sample  N = 4454 | Excluded From the Longitudinal Study Sample  N = 3866 | p-values | η2 |
| Age; M (SD; range) | 65.96 (6.85; 51-95) | 65.94 (7.26; 51-96) | .894 | 0 |
| Women, n (%) | 3475 (78.0) | 2995 (77.5) | .548 |  |
| Education, n (%) |  |  |  |  |
| Secondary education | 592 (13.3) | 538 (13.9) | .534 |  |
| Post-secondary education | 514 (11.4) | 447 (11.6) |  |  |
| Vocational qualification | 899 (20.2) | 745 (19.3) |  |  |
| Undergraduate degree | 1516 (34.0) | 1275 (33.0) |  |  |
| Post-graduate degree | 765 (17.2) | 714 (18.5) |  |  |
| Doctorate | 168 (3.8) | 147 (3.8) |  |  |
| Employed, n (%) | 1737 (40.0) | 1656 (43.9) | <.001 |  |
| Awareness of age-related gains in 2019, M (SD) | 18.00 (3.89) | 17.88 (3.93) | .277 | .0001 |
| Awareness of age-related losses in 2019, M (SD) | 9.76 (3.20) | 10.01 (3.31) | .001 | .001 |
| Attitudes towards own aging in 2019, M (SD) | .68 (.19) | .68 (.19) | .056 | .0004 |
| How frequently do you use any of desktop computer, Laptop, Tablet, Smartphone (iPhone), Smartphone (Android software), Kindle, Wearable fitness monitors (e.g. Fitbit), and Personal music players (e.g. iPod) in 2019 |  |  |  |  |
| Never | 39 (0.9) | 42 (1.1) | .136 |  |
| Occasionally | 8 (0.2) | 14 (0.4) |  |  |
| Once a month | 2 (0.04) | 6 (0.2) |  |  |
| Once a week | 32 (0.7) | 18 (0.5) |  |  |
| Once a day | 334 (7.5) | 291 (7.5) |  |  |
| More than once a day | 4039 (90.7) | 3495 (90.4) |  |  |
| How often do you use…? The internet in 2019 |  |  |  |  |
| Never | 1 (0.02) | 3 (0.1) | .001 |  |
| Occasionally | 0 (0) | 17 (0.4) |  |  |
| Once a month | 5 (0.1) | 1 (0.03) |  |  |
| Once a week | 21 (0.5) | 20 (0.5) |  |  |
| Once a day | 363 (8.2) | 246 (6.4) |  |  |
| More than once a day | 4064 (91.2) | 3579 (92.6) |  |  |
| How often do you use…? Social media (Facebook, Twitter, Instagram, Whatsapp, etc.) in 2019 |  |  |  |  |
| Never | 707 (15.9) | 562 (14.5) | .004 |  |
| Occasionally | 535 (12.0) | 462 (12.0) |  |  |
| Once a month | 31 (0.7) | 23 (0.6) |  |  |
| Once a week | 308 (6.9) | 209 (5.4) |  |  |
| Once a day | 960 (21.6) | 803 (20.8) |  |  |
| More than once a day | 1913 (43.0) | 1807 (46.7) |  |  |
| *Note.* The longitudinal study sample comprises a subsample of participants who reported data on awareness of age-related changes, attitudes towards own aging, and use of social media both in 2019 and 2020. The subsample of participants excluded from longitudinal data did not complete in 2020 one or more measures of awareness of age-related changes, attitudes towards own aging, and use of social media. For eta-squared (η2) effect sizes between 0.01 and 0.05 are interpreted as small, between 0.06 and 0.13 are interpreted as moderate and of 0.14 or above are interpreted as large (Cohen, 1988). | | | | |

| **Supplementary Table 2.**  AARC-gains, AARC-losses, and ATOA as Cross-Sectional Predictors of Frequency of Use of Technologies and the Internet | | | | | | | |
| --- | --- | --- | --- | --- | --- | --- | --- |
| Predictors | **Use of Technologies** | | | | | | |
|  | Unadjusted Model | | | Adjusted Model | | | |
|  | β (95% CI) | p-value | R^2^ | β (95% CI) | p-value | | R^2^ |
| AARC-gains | .03 (.01; .05) | <.001 | 0.1% | .02 (-.0004; .04) | .055 | | 0.04% |
| AARC-losses | -.05 (-.08; -.03) | <.001 | 0.3% | -.03 (-.05; -.01) | .016 | | 0.1% |
| ATOA | .06 (.04; .08) | <.001 | 0.3% | .03 (.01; .06) | .003 | | 0.1% |
|  | **Use of the Internet** | | | | | | |
|  | Unadjusted Model | | | Adjusted Model | | | |
|  | β (95% CI) | p-value | R^2^ | β (95% CI) | p-value | R^2^ | |
| AARC-gains | .03 (.01; .05) | .013 | 0.1% | .03 (.01; .05) | .008 | 0.1% | |
| AARC-losses | -.05 (-.07; -.03) | <.001 | 0.3% | -.03 (-.05; -.01) | .015 | 0.1% | |
| ATOA | .04 (.02; .06) | <.001 | 0.2% | .02 (-.004; .04) | .119 | 0.03% | |
| *Note:* N = 4454. Adjusted for age, sex, education, and employment status | | | | | | | |

| **Supplementary Table 3.**  Interactions Between Age and Each of AARC-gains, AARC-losses, and ATOA as Cross-Sectional Predictors of Frequency of Use of Technologies, the Internet, and Social Media | | | | |
| --- | --- | --- | --- | --- |
| Predictors | **Use of Technologies** | | | |
|  | Unadjusted Model | | Adjusted Model | |
|  | B (95% CI) | p-value | β (95% CI) | p-value |
| AARC-gains x age | .001 (.0001; .001) | .021 | .001 (.001; .001) | .014 |
| AARC-losses x age | -.001 (-.001; -.0002) | .005 | -.001 (-.001; -.0002) | .008 |
| ATOA x age | .01 (-.002; .02) | .122 | .01 (-.003; .02) | .158 |
|  | **Use of the Internet** | | | |
|  | Unadjusted Model | | Adjusted Model | |
|  | B (95% CI) | p-value | β (95% CI) | p-value |
| AARC-gains x age | -.01 (-.03; .01) | .314 | .0002 (-.0001; .0005) | .165 |
| AARC-losses x age | .01 (-.01; .03) | .301 | -.0003 (-.001; .00005) | .097 |
| ATOA x age | -.23 (-.59; .14) | .222 | .01 (.0001; .01) | .045 |
|  | **Use of Social Media** | | | |
|  | Unadjusted Model | | Adjusted Model | |
|  | B (95% CI) | p-value | β (95% CI) | p-value |
| AARC-gains x age | .0004 (-.001; .002) | .562 | .0003 (-.001; .002) | .725 |
| AARC-losses x age | -.003 (-.005; -.002) | <.001 | -.003 (-.01; -.002) | <.001 |
| ATOA x age | .05 (.02; .08) | <.001 | .05 (.02; .08) | .001 |
| *Note.* N = 8320. Adjusted for age, sex, education, and employment status. | | | | |
